# Protease gene families in *Populus *and Arabidopsis

**DOI:** 10.1186/1471-2229-6-30

**Published:** 2006-12-20

**Authors:** Maribel García-Lorenzo, Andreas Sjödin, Stefan Jansson, Christiane Funk

**Affiliations:** 1 Umeå Plant Science Centre, Department of Biochemistry, Umeå University, S – 90187 Umeå, Sweden; 2 Umeå Plant Science Centre, Department of Plant Physiology, Umeå University, S – 90187 Umeå, Sweden

## Abstract

**Background:**

Proteases play key roles in plants, maintaining strict protein quality control and degrading specific sets of proteins in response to diverse environmental and developmental stimuli. Similarities and differences between the proteases expressed in different species may give valuable insights into their physiological roles and evolution.

**Results:**

We have performed a comparative analysis of protease genes in the two sequenced dicot genomes, *Arabidopsis thaliana *and *Populus trichocarpa *by using genes coding for proteases in the MEROPS database [1] for *Arabidopsis *to identify homologous sequences in *Populus*. A multigene-based phylogenetic analysis was performed. Most protease families were found to be larger in *Populus *than in *Arabidopsis*, reflecting recent genome duplication. Detailed studies on e.g. the DegP, Clp, FtsH, Lon, rhomboid and papain-Like protease families showed the pattern of gene family expansion and gene loss was complex. We finally show that different *Populus *tissues express unique suites of protease genes and that the mRNA levels of different classes of proteases change along a developmental gradient.

**Conclusion:**

Recent gene family expansion and contractions have made the *Arabidopsis *and *Populus *complements of proteases different and this, together with expression patterns, gives indications about the roles of the individual gene products or groups of proteases.

## Background

Proteolysis is a poorly understood aspect of plant molecular biology. Although proteases play crucial roles in many important processes in plant cells, e.g. responses to changes in environmental conditions, senescence and cell death, very little information is available on the substrate specificity and physiological roles of the various plant proteases. Even for the most abundant plant protein, ribulose 1,5-bisphosphate carboxylase/oxygenase (Rubisco), neither the proteases involved in its degradation nor the cellular location of the process are known. In the *Arabidopsis thaliana *(hereafter Arabidopsis) genome, many genes with sequence similarities to known proteases have been identified; the MEROPS database (release 7.30) of Arabidopsis proteases contains 676 entries, corresponding to almost 3 % of the proteome. However, protease activity has only been demonstrated for a few of the entries. Most of these putative proteases are found in extended gene families and are likely to have overlapping functions, complicating attempts to dissect the roles of the different proteases in plant metabolism and development.

One scenario in which proteases play a very important role is senescence, although it still is discussed if they actually cause senescence or purely are involved in resource mobilization.

Senescence is the final stage of plant development and can be induced by a number of both external and internal factors such as age, prolonged darkness, plant hormones, biotic or abiotic stress and seasonal responses. An important function of senescence is to reallocate nutrients, nitrogen in particular, to other parts of the plant before the specific structure is degraded. The understanding of senescence is very important for biomass production. In order to understand more about the role of proteases during senescence in this study we compare the nuclear genome of *Arabidopsis thaliana *and *Populus trichocarpa*. The close relationship of these two species in the plant kingdom [2] allows a direct comparison of an annual plant with a tree that has to cope with highly variable adaptations during its long life span. Recent research has shown that leaf senescence affects the chloroplast much earlier than the mitochondria or other compartments of the cell [3], we therefore chose to focus on protease families that express members in this plastid as well as on the papain protease family which consists of proteases that are well-known to be involved in senescence.

In the chloroplast at least 11 different protease families are represented, however, several of them work as processing peptidases. Only 6 families posses members that are known to be involved in degradation, four of these families belong to the class of serine proteases, two are metalloproteases. The Deg proteases form one family (S1, chymotrypsin family) inside the serine clade and the ATP-dependent Clp proteases are grouped in the S14 family. The S16 family contains the so-called Lon proteases. Metalloproteases (MPs) are proteases with a divalent cation cofactor that binds to the active site; most commonly Zn^2+ ^is ligated to two Histidines in the sequence HEXXH. However, Zn^2+ ^can be replaced by Co^2+^, Mn^2+ ^or even Mg^2+^. The M41 family is the group of FtsH proteases and the EGY (ethylene-dependent gravitropism-deficient and yellow-green) proteases belong to the family of S2P proteases (M50).

Comparative genomics analyses could provide valuable insights into the conservation, evolution, abundance and roles of the various plant protease families. For instance, such analyses should facilitate the detection of protein sequences that are conserved in different species, and thus are likely to have common functions in them, and recent expansions of gene families, which should help elucidate issues concerning non-functionalization, neofunctionalization and subfunctionalization. Thus, as reported here, we undertook a comparative analysis of protease gene families in the two sequenced dicot genomes, those of the annual plant Arabidopsis and the tree *Populus trichocarpa *(hereafter *Populus*), with special emphasis on proteases which may play a role in senescence. The results should help to provide a framework for further elucidation of the nature and roles of these complex gene families.

## Results

### Most protease gene families are larger in *Populus *than in *Arabidopsis*

We made an analysis of all protease genes of Arabidopsis and *Populus*. As noted above, conservation of a protein sequence in these two species indicates that it is likely to have a common function in them. Recent expansions of gene families, on the other hand, could provide indications of different adaptive requirements (and, possibly, of more general differences between annual plants and trees).

**Figure 1:**
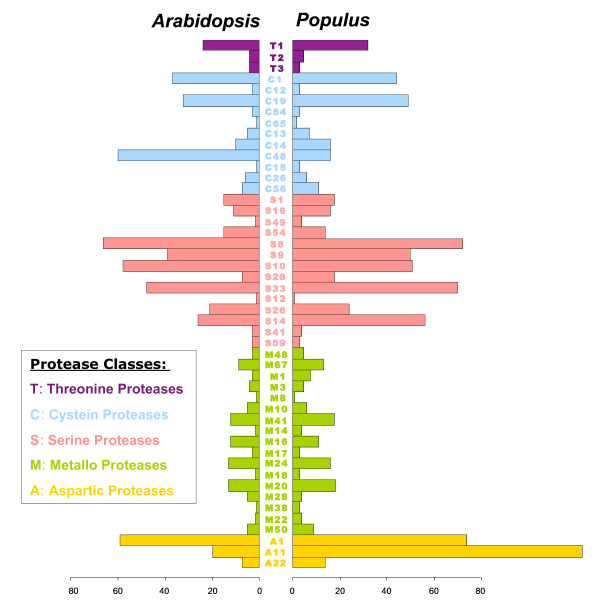
Classification and comparison of proteases in Arabidopsis and *Populus*. The different colors indicate the different protease classes: threonine proteases (T), cysteine proteases (C), serine proteases (S), metalloproteases (M) and aspartic proteases (A). Each class can be divided into different families according to MEROPS, the family number is indicated between the Arabidopsis and *Populus *charts.

The results of the genome comparison between Arabidopsis and *Populus *are compiled in Table 1. In total, we identified 723 genes coding for putative proteases in Arabidopsis and 955 in *Populus*. Forty-five previously unidentified Arabidopsis genes were detected that were not present in the MEROPS database at the time. Like most of the genes in the MEROPS database, we do not know whether or not these genes code for active proteases, but due to their sequence similarity they could have protease activity and were included in the comparison. Figure 1 shows a graphic representation of this comparison. Generally the protease gene numbers in each family do not vary greatly between the two species, although *Populus *has more members in most subfamilies, a consequence of its genome history. Both lineages have undergone rather recent genome duplications [4,5] but the evolutionary clock seems to tick almost six-fold slower in the *Populus *as compared to the Arabidopsis lineage and loss of duplicated genes have been much retarded [4,5]. However, some families were more expanded than others, especially the A11 subfamily of aspartic proteases (the copia transposon endopeptidase family), which has 20 members in Arabidopsis and 123 members in *Populus*. Since the characteristic sequence of these proteases is part of the copia-transposable element, which is abundant in *Populus *[5,6], this expansion is likely to have been simply a consequence of the multiplication of the transposon, rather than selection pressure to increase the copy number of the protease *per se*. Therefore, this family will not be mentioned further. Some subfamilies (the aspartic-type A22, cysteine-type C56, serine-types S49 and S28, and metallo-types M1, M14 and M38) have twice as many members in *Populus *compared to Arabidopsis, but in Arabidopsis these numbers are low, thus duplication could have readily occurred. An interesting case is the subfamily C48, the Ulp1 (ubiquitin-like protease) endopeptidase family, cystein-type, which contains SUMO (small ubiquitin-like modifier) deconjugating enzymes, with 77 members in Arabidopsis, but only 13 in *Populus*. This protein family has been shown to cleave not only the SUMO precursor, but also SUMO ligated to its target proteins; SUMO-ligation probably being involved in many cellular processes, including nuclear export and stress responses [7] and flowering [8]. This family appears to have greatly expanded in Arabidopsis recently.

To confirm the findings described above, case studies were performed in more detail, focusing on proteases that are known to be present in the plant plastids and mitochondria, partly because we have a special interest in organellar biology and partly because these proteases generally belong to the best characterized plant protease families. The "organellar protease subfamilies" chosen for detailed comparisons were: the Deg/HtrA family (chymotrypsin family, S1), Lon protease family (S16), rhomboid protease family (S54) and the Clp endopeptidase family (S14), all belonging to the serine-type class, and the metallo-type FtsH endopeptidase family (M41). In addition, we examined the papain-like cysteine protease family (C1) as certain members are known to play an important role in leaf development, being the necessary machinery that the leaf needs to respond to different kind of stresses or to undergo senescence.

### The FtsH protease family

FtsHs are ATP-dependent proteases that based on the X-ray crystallographic analysis form a homo-oligomeric hexameric ring [9]. *E. coli *FtsH has two transmembrane domains towards the N-terminus that anchor it in the plasma membrane, while the protease domain and the C-terminus face the cytoplasm [10]. Four isomers of FtsH have been identified in *Synechocystis *sp. PCC 6803, 12 in Arabidopsis [11]. Of the nine FtsH that reside in the chloroplast, five have been shown to be involved in the degradation of photosynthetic proteins during light acclimation [12,13] or after high light damage [14-17].

In Arabidopsis the FtsH family is encoded by 16 homologous sequences [11]. Four of these sequences lack the Zn-binding motif and are therefore thought to have lost proteolytic activity. However, they might be involved in chaperone functions instead [18]. In this work we focused on these presumably active proteases. FtsH proteases are thought to be membrane integral, as has been shown experimentally for FtsH1. This protease is inserted into the thylakoid membrane with the Zn-binding and ATPase motifs facing the stroma [14]. Gene comparison studies showed that of the 12 *ftsH *genes potentially coding for fully functional proteases 10 are found in highly homologous pairs. While the pairs AtFtsH1/5, AtFtsH2/8 and AtFtsH 7/9 are targeted to the chloroplast, AtFtsH3/10 and AtFtsH4 have been identified in mitochondria [18,19]. AtFtsH11, which contains only one transmembrane domain was recently suggested to be located in both chloroplasts and mitochondria [19,20]. AtFtsH12 and AtFtsH6, both localized in the chloroplast [12,21] have no pair-partners. The proteins in a pair very likely work in concert, and have overlapping functions as shown for FtsH1/5 and FtsH2/8 [22]. These pairs of proteases are the most strongly expressed FtsHs in plants. Deletion mutants of these genes lead to a variegated leaf type, therefore the names Var1 and Var2 were given to them (reviewed by Sakamoto et al. [21]). The only FtsH protein for which a function has been established, apart from these four proteases, is FtsH6 [13].

**Figure 2:**
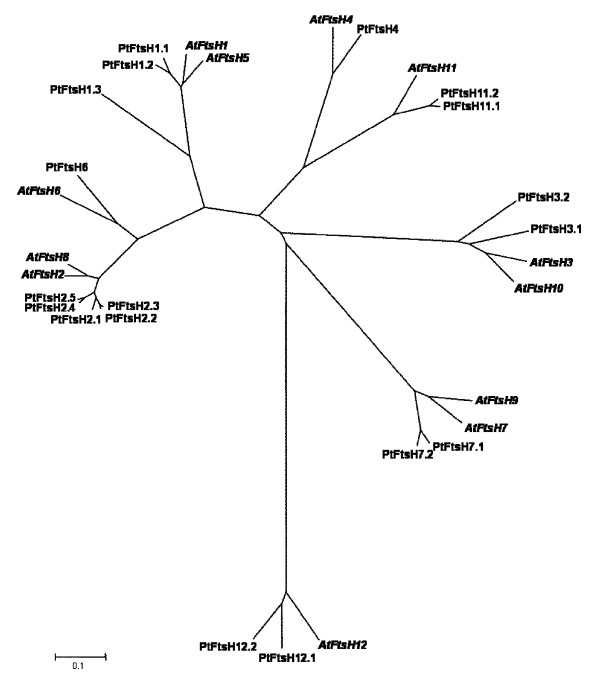
UPGMA (Unweighted Pair Group Method with Arithmetic Mean) tree of the FtsH protease family (M41 family in MEROPS). The names and the accession numbers for the different proteins are given in Table 2.

Figure 2 shows the phylogenetic tree of the *Populus *and Arabidopsis FtsH proteases obtained by Unweighted Pair Group Method with Arithmetic Mean (UPGMA), while their names and accession numbers are given in Table 2. In *Populus*, 16 *ftsH *genes were identified, and in the UPGMA tree, together with the Arabidopsis sequences, we differentiated seven groups, which cluster according to the Arabidopsis FtsH-pairs. When naming the *Populus *genes we tried to follow the Arabidopsis nomenclature. However, in many cases, recent duplications seem to have occurred after the separation of the *Populus *and Arabidopsis lineages and, thus, there are not always clear orthological relationships between the Arabidopsis and *Populus *genes. In such cases, we named the *Populus *genes according to the lowest numbered of the corresponding Arabidopsis pair, e.g. the *Populus *sequences most similar to the AtFtsH3/10 pair were named PtFtsH3.1 and PtFtsH3.2.

The Var2 group, represented by AtFtsH2 and AtFtsH8 in Arabidopsis, has the most *Populus *representatives (PtFtsH2.1, PtFtsH2.2 PtFtsH2.3, PtFtsH2.4 and PtFtsH2.5); all of which are very closely related and appear to have originated from a recent gene family expansion. The Var1 group comprises AtFtsH1, AtFtsH5, PtFtsH1.1 and PtFtsH1.2. A more distant relative of this group is PtFtsH1.3, which has no close Arabidopsis homologue. AtFtsH6 and its *Populus *ortholog, PtFtsH6, are closely related to the Var1/Var2 groups, and clearly separated from the FtsH4/11, FtsH3/10, FtsH7/9 and FtsH12 groups. Interestingly, while in the pairs FtsH1 and 5, FtsH2 and 8, FtsH3 and 10 and FtsH7 and 9 the duplication of the genes seem to have occurred after the separation of *Populus *and Arabidopsis, in the pair FtsH4 and FtsH11 the Arabidopsis proteases have at least one distinct orthologue in *Populus*. Here subfunctionalization seems to have occurred, evident by the fact that AtFtsH4 is found in mitochondria, while AtFtsH11 also can be located in the chloroplast [19,20].

### Some Deg subfamilies are more expanded in Arabidopsis

The Deg proteases form the first family (S1, chymotrypsin family) inside the serine clade. DegP (or HtrA for high temperature requirement) was the first Deg protease identified in *E. coli *[23]. As determined from its crystal structure it functions as homotrimeric oligomer [24], the catalytic center consisting of the residues His-Asp-Ser typical for most serine proteases (SPs). HtrA also functions as a chaperone at low temperature [25]. While cyanobacteria – like *E. coli *– posses 3 members of this family, in the Arabidopsis genome 16 homologues were found. Deg1, 2, 5 and 8 have been identified in the chloroplast [26,27]. In plants and cyanobacteria the Deg proteases are thought to be involved in cell growth, stress responses, PCD and senescence [28,29].

The Deg protease family in Arabidopsis consists of 16 proteins that are localized in different cellular compartments and in many cases have unknown functions. AtDeg1, AtDeg2, AtDeg5 and AtDeg8 are the plastidic members of the AtDeg group. AtDeg1, AtDeg5 and AtDeg8 have been localized in the thylakoid lumen of the plant chloroplast [26,30,31]. AtDeg2 has been identified at the stromal side of the thylakoid membrane and seems, at least in higher plants, to be responsible for the degradation of the reaction center D1 protein of Photosystem II (PSII) [27].

**Figure 3:**
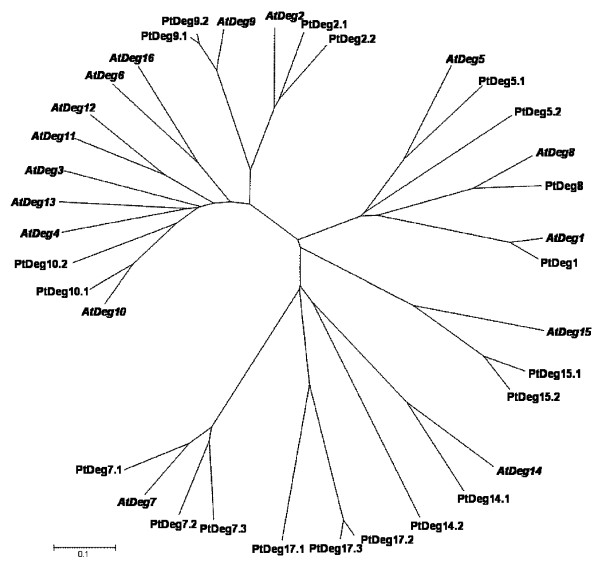
UPGMA (Unweighted Pair Group Method with Arithmetic Mean) tree of the Deg protease family (S1 family in MEROPS). The names and the accession numbers for the different proteins are given in Table 3.

Figure 3 provides an overview of the Deg protease family in Arabidopsis and *Populus*, while Table 3 lists their accession numbers and names. We have identified 20 Deg sequences in *Populus*. In this family some of the Arabidopsis Deg proteases seem to have *Populus *orthologs (Deg1, Deg5, Deg8, Deg14) and often additional, more distantly related *Populus *homologs (Deg5.2, Deg7.2 and Deg7.3, Deg14.2) can be found. In other cases (Deg2, Deg9) two *Populus *sequences are more similar to each other than to the corresponding Arabidopsis protease, indicating a recent gene duplication in *Populus*. The luminal proteases [26] Deg1, 5, and 8 form a clade (Figure 3), indicating a similar function in *Populus *and also the predicted mitochondrial proteases AtDeg3, AtDeg4, AtDeg6, AtDeg10, AtDeg11, AtDeg12, AtDeg13 and AtDeg16 are more closely related. Interestingly only two *Populus *homologs were detected in this group, both of which were most similar to AtDeg10. AtDeg16 (At5g54745) is annotated as a Deg protease in the TAIR database, but has not previously been included in the overview of Arabidopsis proteases [11]. The same is true for AtDeg15 (At1g28320), which has recently been predicted to be localized in peroxisomes [32].

The Deg17 group consists exclusively of *Populus *sequences. These genes code for three proteases that are not closely related to any Arabidopsis protein, but clearly belong to the chymotrypsin family and have a Deg structure, perhaps representing a subfamily that was lost during Arabidopsis evolution (Figure 3).

### The Clp family

Clp proteases are multi-subunit enzymes in which the catalytic domain and the ATPase domain are split in different subunits. Structurally they are very similar to the proteasome 26S in eukaryotes [33]; suggesting that these ATP-dependent proteases are evolutionary related. Proteins in the plant Clp family, consisting of chaperones and proteases involved in the degradation of misfolded proteins [34], have been grouped in two different subclasses [35]. The proteolytically active protease is designated ClpP, but there are also many genes coding for similar proteins lacking the Ser and His amino acid residues of the catalytic triad, and thus representing an inactive form, named ClpR, with unknown function. The regulating subunits work as chaperones that unfold the targeted proteins for degradation, but may also be involved in protein folding independent of proteolysis. Class I chaperones contain two ATP-binding sites like the ClpCs and ClpBs, while the class II chaperones contain only one ATP binding site, like ClpD, ClpF and ClpXs [11,36]. Crystallisation studies [37] have shown that the protease unit, ClpP, forms a tetradecameric barrel-like structure. On one or both ends complexes of ATPase subunits, in *E. coli *either ClpA or ClpX, form homo-hexameric rings. In the absence of ClpP these units can act as chaperones. In chloroplasts, homologues of ClpB and ClpC, but not ClpA form a complex with ClpP [38]. Chloroplast genomes of alga and higher plants contain a gene potentially encoding ClpP and only recently ClpP was also discovered in the nuclear genome [39].

**Figure 4:**
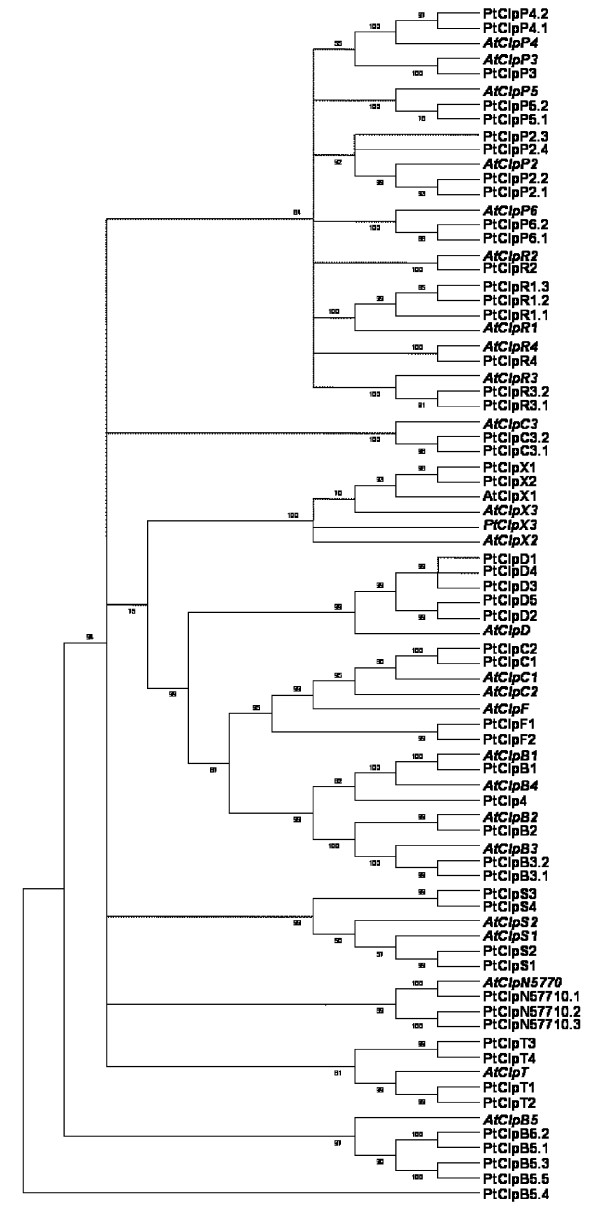
Maximum Parsimony Tree of the Clp protease family (S14 family in MEROPS). The names and the accession numbers for the different proteins are given in Table 4.

We analyzed the homology between Clp proteases in Arabidopsis and *Populus *(Figure 4 and Table 4). In the Maximum Parsimony Phylogenetic Tree (MPT), not surprisingly, a clear separation between the catalytic subunits (ClpP/ClpR) and the regulatory ones can be seen. In the ClpP/ClpR clade, the inactive forms ClpR1, R3 and R4 are more closely related to each other than to the ClpP proteins and the ClpR2. Arabidopsis ClpR1 has three *Populus *homologs, ClpR3 has two and ClpR4 one apparent ortholog.

The ClpR2 sequences from Arabidopis and *Populus *are most similar to the ClpP1 proteins, probably representing a successful case of horizontal gene transfer from the chloroplast to the nucleus that happened before the split of the lineages leading to Arabidopsis and *Populus*. AtClpP1 is encoded in the chloroplast. We found five homologous sequences in the *Populus *nuclear genome, illustrating the flux of genetic material from the chloroplast to the nuclear genome. However, we did not find signs of expression (i.e. associated ESTs) for any of these putative genes, and some of them also appeared not to code for full-length proteins, suggesting that they represent non-functional DNA inserted into the nuclear genome, therefore they will not be further considered here. AtClpP2 has four *Populus *homologs, most of the remaining catalytic AtClp proteins have two or more orthologs in *Populus*, but ClpP3, ClpR2 and ClpR4 each have only one.

The lower part of the MPT in Fig. 4 shows the relationships of the regulatory subunits. Ten well-supported subgroups can be identified: the ClpC3, ClpS, ClpD, ClpC1/C2, ClpF, ClpT, ClpX groups, two ClpB groups, and the ClpN57710 group, containing one Arabidopsis and three *Populus *genes. The separation of the ClpB1-4, ClpC, ClpD and ClpF branches is well supported, with ClpC and ClpF being more closely related to each other than to the other members. The main difference between the ClpD and ClpC groups is that they have specific signature sequences, but they have also been shown to have different expression profiles, ClpDs being specifically expressed in dehydration and senescence [40,41]. The presence of two different ClpB groups is an interesting feature, which can be explained by the fact that At1g07200 (AtClpB5) is grouped by TAIR as a ClpB-related protein. As the nomenclature for ClpB1-4 has already been established, we decided to name this Arabidopsis/*Populus *class ClpB5.

AtClpT is a homolog to the bacterial ClpS, a subunit that in *E. coli *might regulate the activity of the whole Clp complex [42-44]. In *Populus *we find 4 homologs.

Similar to the situation in the other protease families, many Arabidopsis *Clp *genes have two close homologs in *Populus*, but the *ClpD *and *ClpB5 *families are more heavily extended in *Populus*, both having five *Populus *genes compared to a single Arabidopsis gene. There are two *ClpC *members in each organism. However, both of the *Populus ClpC*s seem to be more closely related to *AtClpC1 *than to *AtClpC2*. The ClpX group is predicted to be localized in the mitochondrial matrix in Arabidopsis [11] and it is formed by three proteases in each organism. *AtClpX2 *seems to have a clear ortholog in *Populus*, while the other two *Populus *Cl/pX proteases are more closely related to AtClpX1.

### Lon proteases

Lon proteases (S16 family) are responsible for the degradation of abnormal, damaged and unstable proteins. They have no membrane-spanning domain and contain the AAA (ATPases associated with various cellular activities) and protease domains in one polypeptide. Instead of the Ser-His-Asp of "classical" serine proteases, in Lon proteases the catalytic site is suggested to be formed by a Ser-Lys dyad [45-47]. A crystal structure of Lon in *E. coli *was determined recently and shown to form a hexameric ring [46]. Lon proteases have been described as mitochondrial proteases. However, recent studies have predicted their presence in chloroplasts and peroxisomes [41,48] and Lon4 was shown to be targeted to both chloroplasts and mitochondria [44].

**Figure 5:**
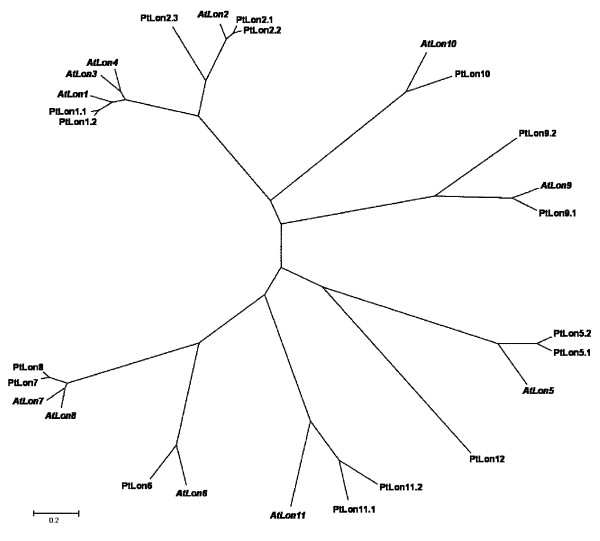
UPGMA (Unweighted Pair Group Method with Arithmetic Mean) tree of the Lon protease family (S16 family in MEROPS). The names and the accession numbers for the different proteins are given in Table 5.

Figure 5 and Table 5 show a phylogenetic comparison of the Lon protease families in Arabidopsis and *Populus*. Except for AtLon1, 3, 4 no subclasses could be detected. However, as for the other families, most Arabidopsis Lon proteases have several orthologs in *Populus*: AtLon1, AtLon2, AtLon5 and AtLon11 are each closely related to a pair of *Populus *orthologs, an apparent result of a recent gene duplication in the tree species. For both AtLon6 and AtLon10 one *Populus *ortholog was found, and the only Arabidopsis Lon proteases that appear to have no *Populus *orthologs are AtLon3 and AtLon4, which are very closely related to each other. One *Populus *sequence, most strongly related to Lon5, did not have a close homolog, and was therefore assigned a name of its own (PtLon12). We have included the Lon9 and Lon10 groups in the Lon family, even though they do not have the ATPase Lon domain. They still belong to the AAA protein family and have some typical Lon protease domains that we considered relevant for the study of this family.

### Rhomboid proteases

The rhomboid family (S54) is a relatively poorly investigated family. It has been widely detected in bacteria, archaea and, recently, eukaryotic organisms – initially in *Drosophila melangolaster *[49,50], then plants [51]. Rhomboid proteases are membrane proteins with six or seven transmembrane domains that cleave their substrates within the substrate's transmembrane domain. This so-called regulated intramembrane proteolysis (RIP) has been shown to be very important for signal transduction. In recent studies of Arabidopsis rhomboids a catalytic dyad has been suggested to be the active site, formed by Ser-His residues [51,52]. The overall structure and sequence of the rhomboid proteases, widely conserved throughout all kingdoms, is very different from that of the other serine proteases, suggesting that they have become serine proteases by convergent evolution [53]. Today, 15 members are annotated in Arabidopsis. Another Arabidopsis gene (*At5g25640*) has high sequence homology to this family, but it is predicted to code for a protein with only two membrane-spanning helices and therefore was not considered in this study. Two rhomboids (AtRbl1 and 2) have been shown to be localized in the Golgi apparatus [52], the subcellular localization of most of the others is predicted to be in mitochondria. Only AtRbl9 and 10 were predicted to be located in the chloroplast using the programs TargetP and Predator. However, the Meta Analysis of the Arabidopsis rhomboid genes in Genevestigator [54] suggests that some of them may play important roles in leaf development and senescence.

**Figure 6:**
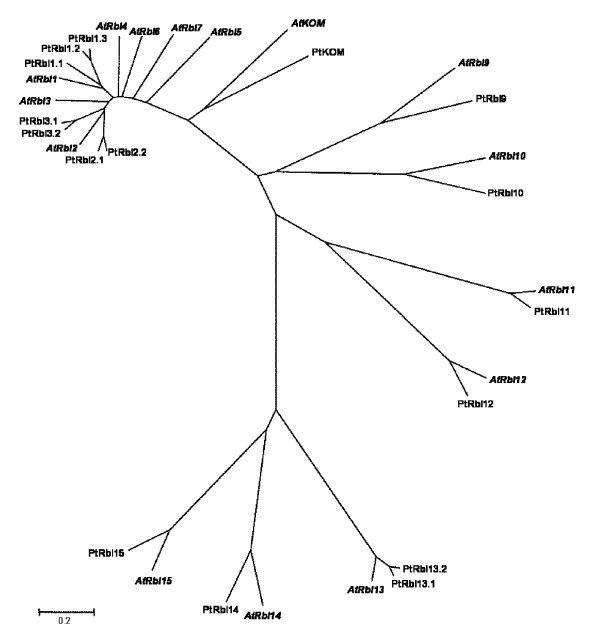
UPGMA (Unweighted Pair Group Method with Arithmetic Mean) tree of the rhomboid protease family (S54 family in MEROPS). The names and the accession numbers for the different proteins are given in Table 6.

Figure 6 shows the comparative UPGMA tree of the rhomboid proteases of Arabidopsis and *Populus*, gene names are explained in Table 6. AtRbl 1–3 are most homologous to rho-1 of *Drosophila melangolaster *and they have 2–3 homologs in *Populus*, as has AtRbl13. The hypothetical plastidic rhomboids AtRbl9 and 10, as well as AtRbl11, AtRbl12, AtRbl14 and AtRbl15 and AtKOM (for kompeitio), each have one clear ortholog in *Populus*. However, AtRbl4 – 7 could not be detected in *Populus*, and these sequences may have evolved after the Arabidopsis-*Populus *divergence.

The EGY proteases belong to the family of S2P proteases (M50), which are ATP-independent metallo-proteases. EGY1 has been recently characterized [55] as a required protease for chloroplast development. With 8 putative transmembrane domains and the intramembrane Zn^2+^-binding domain, these proteases might have a similar structure and function as the rhomboids [44], even though they belong to the class of metalloproteases. The Arabidopsis genome possesses 3 EGYs, EGY1, having been identified in the chloroplast, has one possible orthologue in *Populus*, EGY2 shows homology to one closer and one more distant relative in *Populus*. EGY3 possesses less homology to the other two Arabidopsis proteases and also has one orthologue in *Populus *(not shown).

### Cysteine proteases

In animals, the most representative family of this group is the group of caspases (Cys-Asp-specific proteases, family C14), which play an important role in programmed cell death (PCD) and hypersensitive response (HR) controlling the so-called apoptosis cascade. Closely related proteases in plants are the metacaspases (C14), which have been found to be involved in HR and to act through a caspase-like mechanism [56].

**Figure 7:**
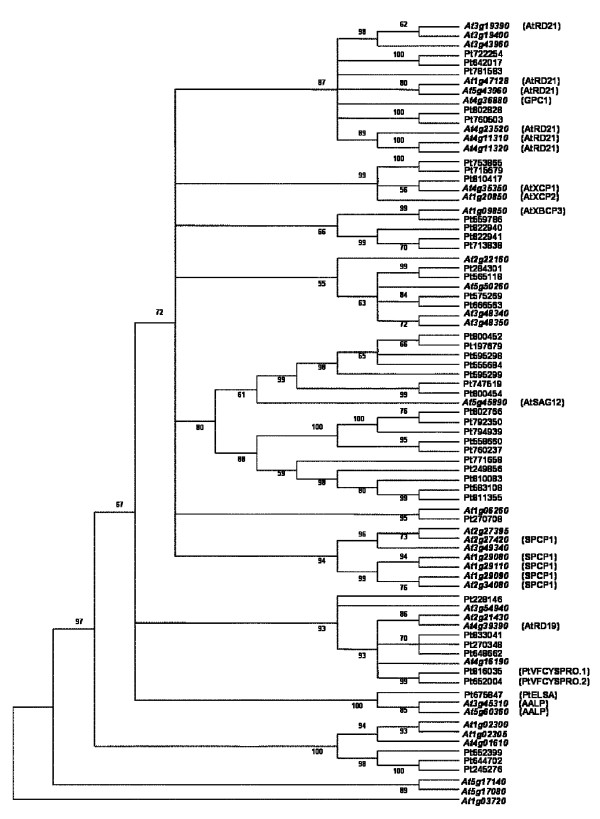
Maximum Parsimony Tree of the papain-like protease family (C1 family in MEROPS). RD, Response to Dehydration; GPC, Germination-specific Cysteine protease; XCP, Xylem Cysteine Protease; XBCP, Xylem and Bark Cysteine Protease; SAG, Senescence-Associated Gene; SPCP, Sweet Potato-like Cysteine Protease; (VFCYSPRO) *Vicia faba *CYStein PROtease; ELSA, Early Leaf-Senescence Abundant cysteine protease; AALP, Arabidopsis Aleurine-Like Protease. The names and the accession numbers for the different proteins are given in Table 7.

The most abundant and thoroughly studied CP family is the papain-like (C1) protease family, which has been related leaf senescence [57-61]. SAG12 (senescence associated gene), the senescence-specific protease [62], is the only protease to be expressed solely during leaf senescence [61] in Arabidopsis and *Brassica napus *[63]. This large family of cysteine proteases also plays diverse roles in defense against pathogens [64]. Thirty-eight papain-like cysteine proteases were identified in Arabidopsis and 44 in *Populus *(Fig. 7, Table 7). The xylem-related cysteine proteases are separated into two different branches, one consisting of the XCPs (xylem cysteine proteases) with two Arabidopsis genes and three *Populus *genes, and the other consisting of the XBCP (xylem and bark cysteine protease) from Arabidopsis with four homologs in *Populus*. The two clades of senescence-related cysteine proteases, including the well-known *SAG12 *genes, consist of many more genes in *Populus *than in Arabidopsis (21 vs. 5). Seven *Populus *proteases have higher homology to the Arabidopsis SAG12 than to any other Arabidopsis proteases, making it difficult to predict if any of these proteases is a functional homolog in *Populus *that plays an essential role during leaf senescence. The second clade consists of 10 *Populus *proteases without any Arabidopsis homologue, indicating the necessity of these proteases in a tree versus an annual plant. However, the RD21 proteases (where RD stands for response to dehydration), that also are known to be involved in senescence, form a separate group, which has more members in Arabidopsis than in *Populus *(nine and five genes, respectively). Also the group containing homologs to SPCP1 (where SCP stands for sweet potato-like cysteine protease) includes seven Arabidopsis genes, but lacks *Populus *representatives.

### Different *Populus *tissues express unique repertoires of proteases

The extensive *Populus *EST resource compiled in PopulusDB [65] allows indications of the expression patterns of *Populus *genes to be rapidly obtained. Of the 951 genes classified above as putative proteases 382 had associated ESTs in PopulusDB, suggesting that these genes, at least, are expressed. Since there are correlations, albeit imperfect, between the abundance of ESTs and the levels of corresponding mRNAs and proteins in particular tissues we wanted to identify the tissues/treatments in which the mRNAs of different types of proteases are most strongly represented. To see if other proteases show similar specificity we examined their digital expression profiles, applying two criteria to reduce the numbers of false positives due to limited information (i.e. the presence of low numbers of ESTs) (table 7). These criteria were (i) more than four ESTs had to be associated with the candidate gene and (ii) more than twice as many ESTs had to be detected in one library than in any other. Only nineteen genes fulfilled these criteria for specific expression. Interestingly, members of the Deg-, FtsH- and papain-like proteases were all highly expressed in senescing leaf tissue. In addition to proteases with particularly high EST frequencies in the senescing leaf and wood cell death libraries, we identified proteases that appeared to be highly expressed in flower buds (four), male catkins (two), the cambial zone (two) and the shoot apical meristem, tension wood, roots and dormant cambium (one in each case). Tissue-specific expression may be the result of a subfunctionalization process, stabilizing both copies of a duplicated gene. To assess the likelihood that such a process has occurred in *Populus*, we sought evidence indicating that unusually high numbers of these genes have undergone recent duplications. We found that the overwhelming majority of the gene families appear to have expanded recently, from one copy in Arabidopsis to two or three copies in *Populus*. This is consistent with the hypothesis that subfunctionalization is one of the forces that has maintained the high proportion of duplicated genes in *Populus*.

**Figure 8:**
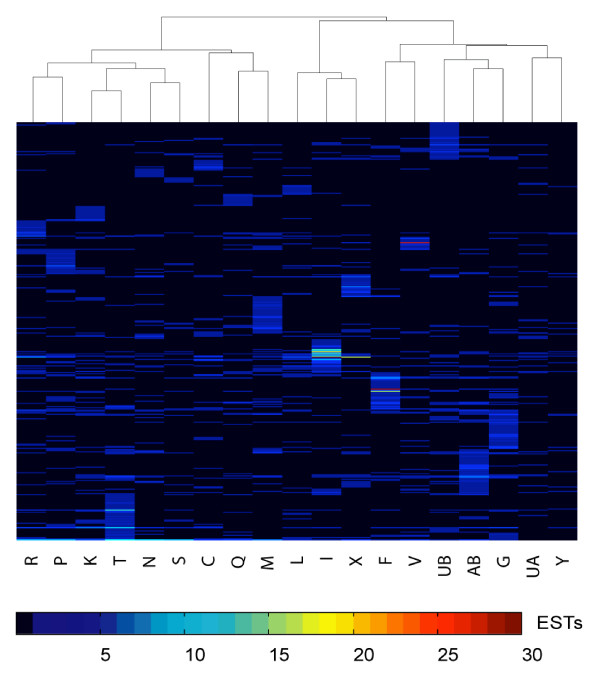
Clustered correlation map of protease EST frequencies across 19 *Populus *cDNA libraries. R: roots, P: petiols, K: apical shoot, T: shoot meristem, N: bark, S: imbibed seeds, C: young leaves, Q: dormant buds, M: female catkins, L: cold-stressed leaves, I: senescing leaves, X: wood cell death, F: floral buds, V: male catkins, UB: active cambium, AB: cambial zone, G: tension wood, UA: dormant cambium, Y: virus/fungal infected leaves. For descriptions of the different libraries, see [65], or [77].

We also constructed a clustered correlation map [66] for all protease genes for which we had EST data. This map (Fig. 8) showed that the different tissues/treatments were associated with quite specific protease expression patterns. Three main clusters could be identified. The senescing leaf library seemed to express a specific set of proteases similar to the wood cell death and the cold-stress leaves libraries, quite distinct from those found in other libraries. But there were also distinct similarities in the patterns of several other libraries, especially the shoot apical meristem, cambial zone, tension wood, flower bud and female flower libraries. Although libraries from similar source material sometimes clustered together (like the cambial zone, tension wood and active cambium libraries), there were also remarkable differences in the repertoire of proteases expressed in similar tissues in some cases, e.g. between active and dormant cambium, and between male and female catkins, which clustered far away from each other. Taken together, this shows that different *Populus *tissues express unique suites of proteases. Most strongly expressed were 8 proteases in the senescing leave library (Fig. 8). The three most strongly transcribed proteases belonged to the papain-like family (RD21, SAG12), followed by proteases with highest similarity to Arabidopsis ClpC, DegP, FtsH8 and FtsH5. The same proteases also had very specific expression in their tissues (table 7).

### Patterns of protease gene expression during Populus leaf development

**Figure 9:**
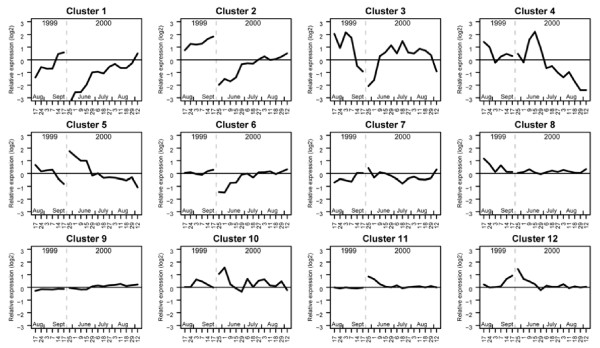
The twelve most common protease expression patterns during *Populus *leaf development. *Populus *DNA microarray data were processed in UPSC-BASE (Sjödin et al. 2006). Samples for microarray analysis were taken from free-growing aspen in Umeå on the following dates; May 25, June 1, June 9, June 15, June 29, July 6, July 18, July 27, August 3, August 11, August 18, August 29 and September 12 2000, and Aug 17, Aug 24, Sept 03, Sept 07, Sept 14 and Sept 17 1999. The two sample series are identified by separate lines in the profiles.

Since we have a particular interest in leaf proteases, we examined the expression of these proteases during *Populus *leaf development in more detail. Over a developmental gradient, it is easy to imagine a number of plausible expression patterns. The simplest may be that some proteases, with functions during leaf expansion, may be expressed in young leaves and their expression levels may gradually decrease, whereas opposite patterns would be expected for others, involved in leaf senescence. Yet others may have different, more complex, patterns. For this analysis, we used two DNA microarray datasets from a mature aspen (*Populus tremula*) grown in the field in Umeå, Sweden [67] (Sjödin et al., submitted). Mature aspens are particularly useful since they only have one flush in the spring, so every leaf at a given date is of the same age, facilitating transcript profiling over a developmental gradient. Bud burst occurs at the end of May and June, and progresses through several phases, during which cell elongation and primary cell wall formation occur, then secondary cell formation peaks. During July and August, no strong trends in gene expression occur and in September, leaf senescence starts [67,68]. We extracted expression profiles for all microarray elements, showing reasonable expression levels some time during leaf development, and performed a hierarchical clustering on the expression profiles (see Additional file 1). As expected, many different patterns were found, but based on the clustering results twelve major patterns were detected. All but three array elements coding for a putative protease exhibited one of these twelve common expression patterns. The expression profiles shown in Figure 9 are representations of these twelve patterns. The two array datasets do not have a common reference, therefore the two expression profiles are separated by a gap in the line. The sampling dates for the first experiment were August 17, August 24, September 3, September 7, September 14, September 17 and September 21, 1999 and the sampling dates for the second series were May 25, June 1, June 9, June 15, June 22, June 29, July 6, July 18, July 27, August 3, August 11, August 18, August 29 and September 12, 2000. Despite these limitations, these data can be used to classify the expression patterns of the leaf proteases.

The genes in cluster 1 are the truly senescence-associated genes. Their mRNA levels did not notably increase until September, but their expression then continued to increase in successive samples, including the last sample from which RNA could be prepared, collected on September 21. This expression pattern was exhibited by genes encoding protease classes C1 (2 genes), C13, C19, M41, M48, S14, S33 (three genes each) and T2 (two genes), i.e. a number of the classes with previously indicated roles during leaf senescence (such as papain-like proteases and FtsH). Cluster 2 had a similar pattern, but the changes were less pronounced, so these genes were only moderately induced during leaf senescence. This cluster contained genes from classes C1, M16, M50, S1, S9 and S14. Cluster 3 consisted of genes that had a fairly stable expression throughout the growing season, but with low mRNA levels during both bud burst and leaf senescence. Pattern 4 was only represented by a S8 (subtilisin) protease gene, which had a pronounced peak during the cell wall biosynthesis phase in the leaf and decreased to low levels in older leaves. Cluster 5 genes were mainly expressed during the first two weeks of leaf development (during the phases mainly characterized by cell division and cell expansion) whereas cluster 6 genes showed the opposite pattern, i.e. they were much more strongly expressed after, rather than during the first two weeks. Cluster 6 was a major cluster, including four genes in the C1 class, seven in the S14 (Clp) class, two in the M1 class, and four other classes. Almost half of the genes coding for proteins in the Clp family appeared to be specifically down regulated when the leaf expanded, suggesting that they have no important function in this stage of leaf development. Clusters 7, 8 and 9 all contain proteases of many different classes, and all showed essentially constitutive expression patterns, except that cluster 7 had lower mRNA levels in the middle of the summer. Clusters 10 and 11, containing mainly serine proteases, both showed high mRNA levels in the first week of leaf development, but cluster 10 seemed to be induced later in the season. Almost all proteasome subunits exhibited expression pattern 11, indicating that the proteasome is most important at the very first stages of aspen leaf development from winter buds. Finally, cluster 12 showed high expression levels only in very young leaves and during late stages of senescence. Taken together, these data indicate that there are several "waves" of protease gene expression during leaf development; consistent with the idea that proteases are important during all stages of the lifecycle of the leaf.

## Discussion

We here present a comparative analysis of the gene families coding for putative proteases of Arabidopsis and *Populus*. The patterns for the copy numbers of most families and subfamilies were quite consistent – the *Populus *families were generally larger, as an apparent result of the fairly recent genome duplication [4,5]. Some families were considerably more heavily represented in *Populus*, but a few were more abundant in Arabidopsis. It seems reasonable to expect, for example, a tree like *Populus *to show relatively strong retention of families like RD21 and SAG12, which are involved in the response to dehydration and leaf senescence, respectively – traits that would intuitively require more elaborate regulation in a tree than in an annual plant, but surprisingly the RD21 family was one of the few gene families that was larger in Arabidopsis than in *Populus*. This supports the view that a considerable element of chance has influenced the size of the gene families in *Populus*, and that stochastic events as well as subfunctionalization and neofunctionalization are important determinants of whether genes are lost or retained in a duplicated genome. Therefore, in most cases, the presence of higher numbers of genes in one plant species than in another cannot be explained simply by their adaptive "needs". However, subfunctionalization and neofunctionalization should not be neglected – in fact, we have shown that they have affected the evolution of the *Populus *genome [69], and our analysis of genes with tissue-specific expression patterns supports this notion.

Unfortunately, of the 723 and 955 proteases identified in Arabidopsis and *Populus*, respectively, the function(s), localization and substrate(s) of most of the proteases remain enigmatic. The Var1/Var2/FtsH6 proteases comprise one of the few protease groups for which mutant phenotypes in Arabidopsis have been carefully examined, and placed in a phylogenetic perspective [13]. Their function in photoprotection seems to have evolved at a very early stage, in the cyanobacterial progenitors of modern cyanobacteria, algae and plants [70]. Later, the Var1 and Var2 functions appear to have separated, and there seems to be an overlap in the substrate specificity of the proteases and the phenotypes of the mutants. *Var1 *and *var2 *are more sensitive than wild type to PSII photoinhibition [15,16]. This duplication of the genes appears to have happened after the separation of Arabidopsis and *Populus *(see Fig. 2). However, in the lineage leading to higher plants, within this group the FtsH6 evolved through neo-functionalization; this protease degrades the antenna rather than reaction center proteins. A clear ortholog of AtFtsH6 can also be found in *Populus*. Based on this very limited information we raise the following hypothesis. If there is a one-to-one relationship between the *Populus *and Arabidopsis sequences, we assume that these genes are functional orthologs, i.e. they degrade the same substrate(s) under the same conditions. However, if the gene duplication happened after the split between Arabidopsis and *Populus *lineages, no neofunctionalization has probably occurred yet, so the functions of these proteases are overlapping. Experiments to verify this hypothesis are in progress.

## Conclusion

Our analysis shows that different tissues express fairly unique sets of genes putatively coding for proteases. Furthermore, in the developmental gradient from bud burst to leaf senescence different waves of protease gene expression occur. However, expression analysis does not always give clear evidence of function. For example, AtFtsH6 has been shown to degrade LHCII only during high light acclimation and senescence [13]; although this protease is essentially constitutively expressed in leaves, its proteolytic activity is regulated by the availability of the substrate. Forward or reverse genetics will be needed to obtain clear information on the involvement of various proteases in different biological processes. However, in order to make reverse genetics efficient, comparative genomics data, such as those presented in this paper, facilitate selection of the best candidates. A simple comparative analysis can provide explanations for experimental data. Since the AtFtsH1/FtsH5 and AtFtsH2/FtsH8 pairs have separated after the split of lineages leading to *Populus *and Arabidopsis, it is not surprising that the pairs will have overlapping and partially redundant functions [71]. This means that mutant analysis, either by forward or reverse genetics, will not always provide clear answers; in many cases, biochemical analysis of protease substrate specificities will probably be needed to assign functions to the individual members of the large protease gene families.

In summary, we have identified 951 genes in the *Populus *genome potentially coding for proteases and comparatively analyzed the protease composition of *Populus *and Arabidopsis.

## Methods

### Database search

The databases searched for annotated proteases were TAIR (The Arabidopsis Information Resource) and TrEMBL (a Computer-annotated supplement to Swiss-Prot). The data were grouped according to the MEROPS protease database families.

Using the TIGR At locus for annotated proteases an ortholog search was performed in the *Populus trichocarpa *database [5,72].

In addition, a *blastp *search was used to collect the *Populus *gene models that were not clustered with any of the Arabidopsis genes. To confirm that these new gene models from *Populus *corresponded to protease genes, a protease-motif search was made in SMART 4.0 [73] and InterProScan [74]. Protein sequences that did not have a typically protease family motif were discarded.

### Protein alignment and Phylogenetic trees

Protein alignment was performed with ClustalX 1.81 [75]. Phylogenetic and molecular evolutionary analyses were conducted using MEGA version 2.1 [76]. The FtsH, Deg, Lon and rhomboid trees were derived using an Unweighted Pair Group Method with Arithmetic Mean (UPGMA) method with 1000 bootstraps. The trees for the Clp and papain-like proteases are Maximum parsimony trees (MPT) with 1000 bootstraps.

All families were analysed with both algorithms, and with several different gap penalties. The choice of trees to display was driven by a desire to keep known or suspected orthologous gene clusters in the same branch of the tree, and to produce figures with size and shape suitable for printing. Trees produced with other algorithms and settings are available on request.

The Arabidopsis nomenclature used in this article follows that proposed by Adam et.al. [41] and further developed by Sokolenko et.al. [11]. As in this nomenclature, protein names were given for *Populus *proteases according to their clustering or proximity in the tree, allowing an intuitive association between the *Populus *proteins and the closest Arabidopsis proteins. We have organized the proteins into groups based on their sequence homology in order to facilitate the new nomenclature proposed for *Populus *proteases.

For the rhomboid proteases in Arabidopsis, we followed the nomenclature initiated by Kanaoka et.al. [52], naming the closest to DmRho-1 (the first rhomboid protease described from *Drosophila melanogaster*) AtRbl1. Since the previously named AtKOM is the 8^th ^member of the family in Kanaoka's article we continued at AtRbl9; higher numbers indicate increasingly distant relationships to DmRho-1.

### Expression analysis

Digital expression profiles were obtained from PopulusDB [77], and analysed in UPSC-BASE  [78] . The similarity between gene models (rows) or cDNA library (columns) expression profiles was estimated according to Ewing et.al. [66] with some modifications. Briefly, similarity between gene models or cDNA library expression profiles was estimated by Pearson's coefficient. From the gene model correlations a pairwise Manhattan distance matrix was calculated and the dendrogram was created with the average agglomeration method. The order of gene models and libraries in their respective dendrograms were used to reorder the original data table. All calculations and plotting were done in the programme language R . [79]

DNA microarray data from Andersson et.al. [67] and Sjödin et al. (submitted) were merged and processed in UPSC-BASE according to the default analysis pipeline  [78] . The normalised data were hierarchical clustered with Euclidean distance and average linkage in the TIGR MultiExperiment Viewer (MeV)  [80] . The dataset were divided into 12 clusters (see Additional file 1) and the average log ratio for each cluster was plotted.

## Authors' contributions

MGL carried out the database searches, sequence alignment, phylogenetic trees performance and drafted the manuscript. AS carried out the expression analysis. SJ and CF conceived of the study, participated in its design and coordination and helped to draft the manuscript.

All authors read and approved the final manuscript.

## Supplementary Material

Additional file 1 Hierarchical clustering of the protease gene expression in *Populus *leaves during the growing season. The microarray dataset is divided in the 12 clusters as depicted as different colors to the right of the figure. The expression data are presented as yellow for up-regulation, black for no difference and blue for down-regulation. Click here for file

## Figures and Tables

**Table 1:** Comparison of numbers of protease genes in Arabidopsis and *Populus*. Families highlighted in bold are those that have been examined in most depth in this study.

**PROTEASE CLASS**	**MEROPS FAMILY**	**FAMILY DESCRIPTION**	**Number of Genes in Arabidopsis**	**Number of Genes in *Populus***
Threonine	T1	Proteasome family	25	32
	T2	Peptidase family T2	4	5
	T3	gamma-glutamyltransferase family	4	3
Cysteine	**C1**	**Papain-like**	38	44
	C12	ubiquitin C-terminal hydrolase family	3	3
	C13	VPE	5	7
	C14	Metacaspases	10	16
	C15	pyroglutamyl peptidase I family	1	3
	C19	ubiquitin-specific protease family	32	49
	C26	gamma-glutamyl hydrolase family	5	4
	C44	Peptidase family C44	8	10
	C48	Ulp1endopeptidase family	77	13
	C54	Aut2 peptidase family	3	3
	C56	PfpI endopeptidase family	5	7
	C65	Peptidase family C65	1	2
Serine	S1	**Chymotrypsin family (Deg)**	16	18
	S8	Subtilisin family	65	72
	S9	Prolyl oligopeptidase family	45	68
	S10	Peptidase family S10	57	51
	S12	D-Ala-D-Ala carboxypeptidase B family	1	1
	**S14**	**ClpP endopeptidase family**	26	53
	**S16**	**Lon protease family**	11	17
	S26	Signal peptidase I family	20	24
	S28	Peptidase family S28	7	18
	S33	Peptidase family S33	51	68
	S41	C-terminal processing peptidase family	3	4
	S49	protease IV family (SppA)	1	3
	**S54**	**Rhomboid family**	15	16
	S59	Peptidase family S59	3	3
Metallo	M1	Peptidase family M1	3	8
	M3	Peptidase family M3	4	5
	M8	leishmanolysin family	1	1
	M10	Peptidase family M10	5	6
	M14	carboxypeptidase A family	2	4
	M16	pitrilysin family	13	11
	M17	leucyl aminopeptidase family	3	3
	M18	Aminopeptidase I	2	3
	M20	Peptidase family M20	13	18
	M22	Peptidase family M22	2	4
	M24	Peptidase family M24	12	16
	M28	Aminopeptidase Y family	5	4
	M38	Beta-aspartyl dipeptidase family	1	3
	**M41**	**FtsH endopeptidase family**	12	18
	M48	Ste24 endopeptidase family	3	5
	M50	S2P protease family	4	5
	M67	Peptidase family M67	9	13
Aspartic	A1	Pepsin-like proteases	59	74
	A11	Copia transposon endopeptidase family	20	123
	A22	presenilin family	8	14

TOTAL			723	955

**Table 2:** Arabidopsis (At) and *Populus *(Pt) FtsH protease gene models (M41 family in MEROPS) corresponding to the names given in the FtsH phylogenetic tree.

**Group**	**At name**	**At number**	***Populus *Gene model**	**Pt number**	**Pt name**
Var1	AtFtsH5	At5g42270	gw1.II.2305.1	Pt421671	PtFtsH5.1
	AtFtsH1	At1g50250	gw1.V.2026.1	Pt206625	PtFtsH5.2
			gw1.16150.2.1	Pt273866	PtFtsH5.3
Var2	AtFtsH8	At1g06430	gw1.XIV.2894.1	Pt246151	PtFtsH8.1
	AtFtsH2	At2g30950	estExt_fgenesh4_pg.C_3210002	Pt828819	PtFtsH8.2
			eugene3.17410001	Pt585288	PtFtsH8.3
			eugene3.00001972	Pt552657	PtFtsH8.4
			gw1.321.23.1	Pt284497	PtFtsH8.5
H3	AtFtsH3	At2g29080	fgenesh4_pm.C_LG_IX000602	Pt804555	PtFtsH3.1
	AtFtsH10	At1g07510	fgenesh4_pm.C_LG_XVI000360	Pt808632	PtFtsH3.2
H4	AtFtsH4	At2g26140	gw1.VI.123.1	Pt426451	PtFtsH4
H6	AtFtsH6	At5g15250	fgenesh4_pg.C_LG_XVII000398	Pt778519	PtFtsH6
H7	AtFtsH7	At3g47060	gw1.IX.3866.1	Pt203401	PtFtsH7.1
	AtFtsH9	At5g58870	gw1.I.994.1	Pt172394	PtFtsH7.2
H11	AtFtsH11	At5g53170	estExt_fgenesh4_pg.C_LG_XII0132	Pt823192	PtFtsH11.1
			gw1.XV.551.1	Pt251115	PtFtsH11.2
H12	AtFtsH12	At1g79560	eugene3.00101628	Pt567070	PtFtsH12.1
			eugene3.00080778	Pt564183	PtFtsH12.2

**Table 3:** Arabidopsis (At) and *Populus *(Pt) Deg protease gene models (S1 family in MEROPS) corresponding to the names given in the Deg phylogenetic tree.

**Group**	**At name**	**At number**	***Populus *Gene model**	**Pt number**	**Pt name**
Deg1	AtDeg1	At3g27925	estExt_Genewise1_v1.C_LG_I2430	Pt706718	PtDeg1
Deg2	AtDeg2	At2g47940	eugene3.00140795	Pt572750	PtDeg2.1
			fgenesh4_pg.C_LG_XIV001476	Pt775566	PtDeg2.2
Deg5	AtDeg5	At4g18370	fgenesh4_pg.C_LG_XI000444	Pt771291	PtDeg5.1
			fgenesh4_pg.C_scaffold_3341000001	Pt792125	PtDeg5.2
Deg7	AtDeg7	At3g03380	estExt_fgenesh4_pg.C_LG_II2234	Pt816849	PtDeg7.1
			eugene3.00040664	Pt555951	PtDeg7.2
			estExt_Genewise1_v1.C_LG_IV3539	Pt714140	PtDeg7.3
Deg8	AtDeg8	At5g39830	gw1.IV.4356.1	Pt199267	PtDeg8
Deg9	AtDeg9	At5g40200	gw1.XV.1425.1	Pt251989	PtDeg9.1
			estExt_Genewise1_v1.C_LG_XII1032	Pt728836	PtDeg9.2
Deg10	AtDeg3	At1g65630			
	AtDeg4	At1g65640			
	AtDeg6	At1g51150			
	AtDeg13	At5g40560			
	AtDeg12	At3g16550			
	AtDeg11	At3g16540			
	AtDeg10	At5g36950	gw1.VIII.1400.1	Pt430673	PtDeg10.1
			eugene3.00101698	Pt567140	PtDeg10.2
	AtDeg16	At5g54745			
Deg14	AtDeg14	At5g27660	grail3.0016016001	Pt662713	PtDeg14.1
			grail3.0016016101	Pt662714	PtDeg14.2
Deg15	AtDeg15	At1g28320	eugene3.00040486	Pt555773	PtDeg15.1
			gw1.124.194.1	Pt266544	PtDeg15.2
Deg17			fgenesh4_pg.C_scaffold_193000050	Pt787034	PtDeg17.1
			eugene3.01930055	Pt586371	PtDeg17.2
			eugene3.00180012	Pt577788	PtDeg17.3

**Table 4:** Arabidopsis (At) and *Populus *(Pt) Clp protease gene models (S14 family in MEROPS) corresponding to the names given in the Clp phylogenetic tree.

**Group**	**At name**	**At number**	***Populus *Gene model**	**Pt number**	**Pt name**
ClpB	AtClpB1	At1g74310	estExt_Genewise1_v1.C_820051	Pt742398	PtClpB1
	AtClpB2	At2g25140	estExt_Genewise1_v1.C_LG_VI2692	Pt717883	PtClpB2
	AtClpB3	At5g15450	fgenesh4_pg.C_scaffold_3401000001	Pt792165	PtClpB3.1
			eugene3.00041061	Pt556348	PtClpB3.2
	AtClpB4	At4g14670	fgenesh4_pg.C_LG_XVII000457	Pt778578	PtClpB4
	AtClpB5	At1g07200	gw1.I.864.1	Pt172264	PtClpB5.1
			estExt_fgenesh4_pm.C_LG_IX0543	Pt833234	PtClpB5.2
			grail3.0022012901	Pt659508	PtClpB5.3
			grail3.0020020101	Pt669488	PtClpB5.4
			grail3.0010001601	Pt656256	PtClpB5.5
ClpC	AtClpC1	At5g50920	eugene3.00120993	Pt570340	PtClpC1
	AtClpC2	At3g48870	eugene3.00150843	Pt575448	PtClpC2
	AtClpC3	At3g53270	gw1.278.9.1	Pt281354	PtClpC3.1
			gw1.VI.1596.1	Pt427924	PtClpC3.2
ClpD	AtClpD	At5g51070	fgenesh4_pg.C_LG_XII001082	Pt773307	PtClpD1
			eugene3.00150893	Pt575498	PtClpD2
			fgenesh4_pg.C_LG_XII001084	Pt773309	PtClpD3
			fgenesh4_pg.C_scaffold_232000029	Pt787878	PtClpD4
			fgenesh4_pg.C_scaffold_15088000001	Pt794999	PtClpD5
ClpF	AtClpF	At3g45450	fgenesh4_pg.C_scaffold_14521000001	Pt794891	PtClpF1
			fgenesh4_pg.C_LG_V001142	Pt761090	PtClpF2
	AtClpN57710	At5g57710	grail3.0030025301	Pt653660	PtClpN57710.1
			fgenesh4_pg.C_LG_X002263	Pt770773	PtClpN57710.2
			eugene3.00080144	Pt563549	PtClpN57710.3
ClpP	AtClpP2	At5g23140	grail3.0026027701	Pt650895	PtClpP2.1
			eugene3.00070756	Pt562818	PtClpP2.2
			eugene3.33100002	Pt590732	PtClpP2.3
			grail3.4268000201	Pt678327	PtClpP2.4
	AtClpP3	At1g66670	gw1.IV.3459.1	Pt198370	PtClpP3
	AtClpP4	At5g45390	eugene3.00030757	Pt554124	PtClpP4.1
			gw1.29.348.1	Pt434537	PtClpP4.2
	AtClpP5	At1g02560	estExt_fgenesh4_pm.C_LG_II0893	Pt830458	PtClpP5.1
			estExt_Genewise1_v1.C_LG_XIV2274	Pt731676	PtClpP5.2
	AtClpP6	At1g11750	estExt_Genewise1_v1.C_LG_IV0459	Pt712936	PtClpP6.1
			estExt_fgenesh4_pg.C_LG_IX0507	Pt821196	PtClpP6.2
ClpR	AtClpR1	At1g49970	estExt_fgenesh4_pg.C_LG_IX0730	Pt821289	PtClpR1.1
			gw1.I.4091.1	Pt175491	PtClpR1.2
			eugene3.16840002	Pt584851	PtClpR1.3
	AtClpR2	At1g12410	estExt_fgenesh4_pg.C_1270005	Pt827867	PtClpR2
	AtClpR3	At1g09130	gw1.XIII.856.1	Pt240607	PtClpR3.1
			eugene3.01330032	Pt581876	PtClpR3.2
	AtClpR4	At4g17040	eugene3.01180098	Pt580163	PtClpR4
ClpS	AtClpS1	At4g25370	fgenesh4_pg.C_LG_XV001031	Pt776603	PtClpS1
	AtClpS2	At4g12060	fgenesh4_pg.C_LG_XII001246	Pt773471	PtClpS2
			gw1.127.5.1	Pt266999	PtClpS3
			gw1.I.9317.1	Pt180717	PtClpS4
ClpT	AtClpT	At1g68660	estExt_fgenesh4_pg.C_LG_X1165	Pt822150	PtClpT1
			grail3.0010047002	Pt656784	PtClpT2
			estExt_fgenesh4_pg.C_LG_VIII1289	Pt820724	PtClpT3
			estExt_fgenesh4_pg.C_LG_X0879	Pt822021	PtClpT4
ClpX	AtClpX1	At5g53350	gw1.XV.374.1	Pt250938	PtClpX1
	AtClpX2	At5g49840	gw1.XII.172.1	Pt432413	PtClpX2
	AtClpX3	At1g33360	gw1.86.193.1	Pt297302	PtClpX3

**Table 5:** Arabidopsis (At) and *Populus *(Pt) Lon protease gene models (S16 family in MEROPS) corresponding to the names given in the Lon phylogenetic tree.

**Group**	**At Name**	**At number**	***Populus *Gene model**	**Pt number**	**Pt Name**
Lon1	AtLon1	At5g26860	gw1.XIII.616.1	Pt240367	PtLon1.1
			gw1.133.222.1	Pt268780	PtLon1.2
Lon2	AtLon2	At5g47040	estExt_fgenesh4_pg.C_1180067	Pt827676	PtLon2.1
			gw1.12936.1.1	Pt267629	PtLon2.2
			estExt_fgenesh4_pm.C_290060	Pt836320	PtLon2.3
	AtLon3	At3g05780			
	AtLon4	At3g05790			
Lon5	AtLon5	At2g25740	estExt_fgenesh4_pg.C_LG_XVIII0237	Pt825668	PtLon5.1
			estExt_fgenesh4_pg.C_LG_VI1620	Pt819532	PtLon5.2
Lon6	AtLon6	At1g18660	fgenesh4_pg.C_LG_XII000664	Pt772889	PtLon6
Lon7	AtLon7	At1g19740	gw1.V.1534.1	Pt206133	PtLon7
	AtLon8	At1g75460	fgenesh4_pm.C_LG_II000142	Pt798453	PtLon8
Lon9	AtLon9	At2g03670	fgenesh4_pm.C_scaffold_29000155	Pt813379	PtLon9.1
			estExt_fgenesh4_pg.C_LG_XV0552	Pt824692	PtLon9.2
Lon10	AtLon10	At1g73170	gw1.I.4975.1	Pt176375	PtLon10
Lon11	AtLon11	At1g35340	Eugene3.00410149	Pt592306	PtLon11.1
			Eugene3.00190704	Pt574230	PtLon11.2
Lon12			fgenesh4_pg.C_scaffold_3310000001	Pt792107	PtLon12

**Table 6:** Arabidopsis (At) and *Populus *(Pt) rhomboid protease gene models (S54 family in MEROPS) corresponding to the names given in the Lon phylogenetic tree.

**At name**	**At number**	***Populus *Gene model**	**Pt number**	**Pt name**
AtRbl1	At2g29050	gw1.VI.164.1	Pt426492	PtRbl1.1
		estExt_Genewise1_v1.C_LG_I1244	Pt706133	PtRbl1.2
		gw1.IX.4200.1	Pt203735	PtRbl1.3
AtRbl2	At1g63120	estExt_fgenesh4_pm.C_LG_III0384	Pt830825	PtRbl2.1
		estExt_fgenesh4_pg.C_LG_I0956	Pt815105	PtRbl2.2
AtRbl3	At5g07250	gw1.XII.335.1	Pt432576	PtRbl3.1
		estExt_fgenesh4_pg.C_LG_XV1114	Pt824920	PtRbl3.2
AtRbl4	At3g53780			
AtRbl5	At1g52580			
AtRbl6	At1g12750			
AtRbl7	At4g23070			
AtKOM	At1g77860	fgenesh4_pg.C_LG_II000834	Pt754654	PtKOM
AtRbl9	At5g38510	eugene3.01230069	Pt580779	PtRbl9
AtRbl10	At1g25290	estExt_fgenesh4_pg.C_LG_III0079	Pt817004	PtRbl10
AtRbl11	At5g25752	gw1.XVIII.1336.1	Pt260795	PtRbl11
AtRbl12	At1g18600	gw1.VI.85.1	Pt426413	PrRbl12
AtRbl13	At3g59520	grail3.0064008701	Pt679599	PtRbl13.1
		eugene3.00070158	Pt562220	PtRbl13.2
AtRbl14	At3g17611	grail3.0102004101	Pt657794	PtRbl14
AtRbl15	At3g58460	estExt_fgenesh4_pm.C_LG_VI0468	Pt831984	PtRbl15

**Table 7:** *Populus *gene models whose ESTs are specific to a unique library and comparative numbers of the corresponding genes in Arabidopsis. Libraries: (I) senescing leaves, (F) flower buds, (T) shoot meristem, (V) male catkins, (AB) cambial zone, (UB) active cambium, (G) tension wood, (X) wood cell death.

**ProteinNr**	**Unique library**	**Annotation**	**Number of Genes in family of Arabidopsis**	**Number of Genes in family of *Populus***
Pt816035	I	PtVFCYSPRO.1	1	2
Pt814139	F	Proteasome subunit beta type 2-2	1	3
Pt781583	I	RD21 Papain-Like cysteine protease	9	5
Pt678915	T	Proteasome subunit beta type 2-2	1	3
Pt666563	V	PtCYSP2.1	4	4
Pt722254	I	RD21 Papain-Like cysteine protease	9	5
Pt721246	F	aminoacylase	1	2
Pt830360	F	20S proteasome alpha subunit F	1	2
Pt717215	AB	Proteasome subunit alpha type 6-1	1	2
Pt417380	UB	aminopeptidase M	1	5
Pt419163	F	20S proteasome beta subunit.	1	2
Pt713305	V	Proteasome subunit	1	3
Pt747519	I	similar to SAG12	1	7
Pt819223	G	Metallopeptidase M24 family protein	1	3
Pt706718	I	PtDeg1	1	1
Pt410970	I	PtFtsH5.1	1	3
Pt585288	I	PtFtsH8.3	1	3
Pt709916	X	subtilase family protein	1	3
Pt559264	AB	Proteasome subunit beta type 3-2	2	2
