# Supplementary material for: Protease gene families in Populus and Arabidopsis
Source: BMC Plant Biol. 2006 Dec 20;6:30. doi: 10.1186/1471-2229-6-30 (PMC1780054; doi:10.1186/1471-2229-6-30)

**Additional file 1.** Hierarchical clustering of the protease gene expression in *Populus* leaves during the growing season


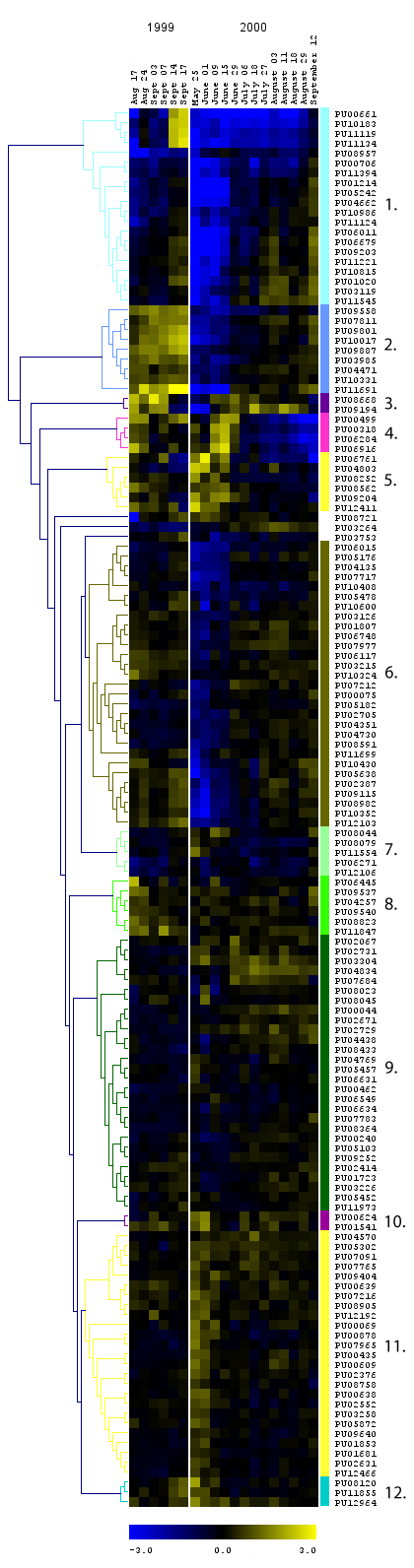

Supplement: Additional file 1 — Hierarchical clustering of the protease gene expression in Populus leaves during the growing season. The microarray dataset is divided in the 12 clusters as depicted as different colors to the right of the figure. The expression data are presented as yellow for up-regulation, black for no difference and blue for down-regulation. [file 1471-2229-6-30-S1.doc]
